# Defining the Prognostic Significance of BRAF V600E in Early-Stage Colon Cancer: A Systematic Review and Meta-Analysis

**DOI:** 10.3390/curroncol32110624

**Published:** 2025-11-06

**Authors:** Matthew Dankner, Laurie-Rose Dubé, Mark Sorin, Andrew J. B. Stein, Alexander Nowakowski, Changsu Lawrence Park, Jamie Magrill, Anna-Maria Lazaratos, Joan Miguel Romero, Gerald Batist, Petr Kavan, April A. N. Rose, Kim Ma

**Affiliations:** 1 Faculty of Medicine, McGill University, Montreal, QC H3A 0G4, Canada; matthew.dankner@mail.mcgill.ca (M.D.); laurie-rose.dube@mail.mcgill.ca (L.-R.D.); mark.sorin@mail.mcgill.ca (M.S.); andrew.stein2@mail.mcgill.ca (A.J.B.S.); alexander.nowakowski@mail.mcgill.ca (A.N.); jamie.magrill@mail.mcgill.ca (J.M.); joan.romero@mail.mcgill.ca (J.M.R.); gerald.batist@mcgill.ca (G.B.); petr.kavan@mcgill.ca (P.K.); april.rose@mcgill.ca (A.A.N.R.); 2 Rosalind and Morris Goodman Cancer Institute, Montreal, QC H3T 1E2, Canada; anna-maria.lazaratos@mail.mcgill.ca; 3 Lady Davis Institute for Medical Research & Segal Cancer Centre, Jewish General Hospital, Montréal, QC H3T 1E2, Canada; 4 Gerald Bronfman Department of Oncology, McGill University, Montréal, QC H3A 0G4, Canada; 5 Faculty of Medicine, Université de Montréal, Montreal, QC H3C 3J7, Canada

**Keywords:** BRAF, encorafenib, colon cancer, adjuvant, targeted therapy

## Abstract

**Simple Summary:**

BRAF mutations occur in 10% of colon cancers and are linked to poor outcomes in metastatic disease. While cancers with BRAF V600E mutations respond well to cetuximab + encorafenib in the metastatic setting, its impact in early-stage colon cancer needs to be established as new trials are testing encorafenib-based treatments in this population. We conducted a systematic review and meta-analysis of six randomized controlled trials involving 6836 patients with BRAF wild-type and 843 patients with BRAF V600E mutations in stage 2–3 colon cancer. We analyzed overall survival and disease-free survival. Results showed that BRAF V600E mutations were associated with significantly worse overall survival and disease-free survival compared to wild-type BRAF. This poor prognosis pattern remained even in patients with microsatellite stable tumors. These findings confirm that BRAF V600E mutations predict inferior outcomes in early-stage colon cancer, providing crucial information for designing future clinical trials targeting this specific patient population.

**Abstract:**

Background: BRAF mutations are found in 10% of colon cancers (CCs) and are associated with poor prognosis in metastatic disease. BRAF V600E predicts sensitivity to cetuximab + encorafenib in the metastatic setting. With new trials testing encorafenib-containing regimens for early-stage CC, we sought to characterize the clinical outcomes of early-stage BRAF V600E CC. Methods: We performed a systematic review and meta-analysis. Key inclusion criteria were a diagnosis of stage 2/3 BRAF V600E CC. Co-primary endpoints were overall survival (OS) and recurrence/disease-free survival (DFS). Meta-analysis was performed with a random-effects model incorporating sample size, hazard ratio (HR), and 95% confidence intervals (CIs). Results: A total of 206 studies underwent full-text review. Of these, six randomized controlled trials were included, comprising 6836 and 843 patients with wild-type (WT) and BRAF V600E, respectively. BRAF V600E was associated with inferior OS (HR 1.49, CI 1.21–1.75) and DFS (HR 1.17, CI 1.03–1.33). This finding remains in patients with microsatellite instability—low/stable or proficient mismatch repair (OS: HR 1.66, CI 1.36–2.02, DFS: HR 1.45, CI 1.22–1.72). Conclusions: BRAF V600E is associated with inferior prognoses compared to BRAF WT in early-stage CC. This finding will help optimize trial design for this population.

## 1. Introduction

BRAF is a constituent of the MAP Kinase (MAPK) signaling pathway and is among the most commonly mutated genes in human cancers [1]. BRAF mutations are found in 10% of colorectal cancers (CRCs) and can be grouped into three classes based on their molecular characteristics [2,3]. Class 1 BRAF (V600) mutations comprise the majority of oncogenic BRAF mutations in most cancer types, including 90% of BRAF mutations found in CRC [3]. This contrasts with Class 2 and 3 non-V600 BRAF mutations, which make up 10% of the BRAF mutations found in CRC [1]. Patients with CRC whose tumors harbor Class 2 and 3 BRAF mutations have been shown to have prolonged survival compared to those with Class 1 BRAF mutations, but are generally believed to be less responsive to MAPK-targeted therapies compared to Class 1 BRAF-mutant CRC [4,5].

Small-molecule inhibitors of BRAF (vemurafenib, dabrafenib, encorafenib) were developed and first studied in BRAF V600-mutant melanoma, demonstrating impressive overall response rates (ORRs) but short progression-free survival (PFS) due to rapid MAPK-pathway reactivation [6,7,8]. Combinations of BRAF inhibitors with MEK inhibitors (cobimetinib, trametinib, binimetinib) successfully prolonged PFS and ultimately overall survival (OS) compared to BRAF-inhibitor monotherapy [9,10]. In metastatic CRC with BRAF V600E mutations, early studies demonstrated that single-agent BRAF inhibition had unimpressive clinical activity, with ORRs of 0–5% [11,12]. This stark contrast between CRC and other BRAF-mutated cancer types has been shown to be related to EGFR-mediated reactivation of the MAPK pathway upon inhibition of BRAF [13,14]. Subsequent studies using EGFR and BRAF inhibition with vemurafenib and cetuximab in Class 1 BRAF-mutant CRC revealed mild improvements in ORR, but still did achieve the clinical efficacy of single-agent BRAF inhibition in other BRAF-mutated cancer types [15,16]. MAPK-targeted approaches did not become an important part of the treatment armamentarium for metastatic colorectal cancer with BRAF V600E until the presentation of data from the BEACON trial [17].

The BEACON trial randomized patients with BRAF V600E-mutant metastatic colorectal cancer who had disease progression after 1–2 previous lines of therapy to receive encorafenib, binimetinib, and cetuximab (triplet therapy group); encorafenib and cetuximab (doublet therapy group); or the investigators’ choice of either cetuximab and irinotecan or cetuximab and FOLFIRI [17]. While the triplet therapy elicited an ORR of 27% and OS of 9.3 months, both statistically superior to the control group, only ORR was numerically superior in the triplet compared to the doublet groups (ORR 19.5%), and OS was identical (9.3 months) [18]. This established encorafenib-and-cetuximab doublet therapy as the standard of care for previously treated BRAF V600E-mutant metastatic CRC.

Subsequently, the phase 2 ANCHOR CRC study demonstrated an ORR of 47% in metastatic CRC with BRAF V600E treated in the first line with the encorafenib, binimetinib, and cetuximab triplet, suggesting that moving MAPK-targeted therapies to the first line may yield benefits for these patients [19]. Indeed, the phase 3 BREAKWATER trial assessed the efficacy of encorafenib and cetuximab with mFOLFOX6 chemotherapy, compared to investigators’ choice standard-of-care (SOC) chemotherapy (FOLFOXIRI, CAPOX or mFOLFOX6) with or without bevacizumab [20]. Results from the trial demonstrated an ORR of 61% with encorafenib, cetuximab, and chemotherapy in the first line, compared to 40% in the SOC arm [20]. OS was also significantly prolonged compared to SOC [20], which has led to accelerated approval by the FDA for encorafenib and cetuximab with mFOLFOX6 in the first-line setting for metastatic CRC with BRAF V600E.

MAPK-targeted therapy strategies are being actively trialed in the adjuvant setting for early-stage disease. The single-arm phase 2 TRESBIEN study plans to treat early recurrent stage 2/3 BRAF V600E-mutant CRC with encorafenib, binimetinib, and cetuximab [21]. Meanwhile, the randomized phase 2/3 study A022004 aims to treat patients with encorafenib and cetuximab versus usual care in the adjuvant setting for patients with stage 2B and stage 3 colon cancer (CC) with BRAF V600E [22]. Together, these studies will explore bringing MAPK-targeted therapy forward in the treatment of these cancers. Ongoing research in liquid biopsy is poised to allow for non-invasive monitoring of disease progression and improved understanding of resistance mechanisms in the context of MAPK-targeted therapies [23].

While it has been clearly established that BRAF V600E is a poor prognostic indicator in metastatic CRC [24,25,26], mixed data exists for early-stage disease. In terms of disease-free survival (DFS), the PETACC-8 and PETACC-3 studies showed no difference between BRAF-mutant and wild-type (WT) patients [27,28]. However, both PETACC-8 and PETACC-3 show inferior overall survival in BRAF-mutant patients, while other studies such as MOSAIC do not [27,28,29]. To evaluate whether BRAF V600E represents a prognostic biomarker in early-stage CC, we performed a systematic review and meta-analysis in anticipation of the forthcoming trial data that aims to improve the outcomes of these patients with MAPK-targeted therapies.

## 2. Materials and Methods

### 2.1. Search Strategy

A literature search was conducted of the studies published from 1 January 2005 to 6 August 2023 in MEDLINE (2005 to 6 August 2023) and EMBASE (2005 to 6 August 2023) using the search strategies specified below. All searches were performed on 6 August 2023. Published conference abstracts were included. The AACR, ASCO, and ESMO conference proceedings were searched to identify any relevant conference abstracts. The study protocol was prospectively uploaded to PROSPERO (ID: CRD42023452221) and followed the Preferred Reporting Items for Systematic Reviews and Meta-Analyses (PRISMA) guidelines [30].

The following search strategies were employed:

MEDLINE (via PubMed): (colon cancer or colon carcinoma or colorectal cancer or colorectal carcinoma or rectal cancer or rectal carcinoma or colorectal neoplasms or rectal neoplasms or bowel neoplasms or colon neoplasms or large intestine cancer or large intestine carcinoma or bowel cancer or bowel carcinoma or CRC) and (BRAF or B-RAF) and (prognosis or outcome or survival or resection or surgical procedures, operative or adjuvants, pharmaceutic or neoadjuvant therapy).

EMBASE (via OVID): (‘colon cancer’ OR ‘colon carcinoma’ OR ‘colorectal cancer’ OR ‘colorectal carcinoma’ OR ‘rectal cancer’ OR ‘rectal carcinoma’ OR ‘colorectal neoplasms’ OR ‘rectal neoplasms’ OR ‘bowel neoplasms’ OR ‘colon neoplasms’ OR ‘large intestine cancer’ OR ‘large intestine carcinoma’ OR ‘bowel cancer’ OR ‘bowel carcinoma’ OR ‘CRC’) AND (BRAF OR B-RAF) AND (prognosis OR outcome OR survival OR resection OR surgical procedures, operative OR adjuvants, pharmaceutic OR neoadjuvant therapy).

### 2.2. Deduplication of Search Results

Deduplication was performed using the synthesiser package (v0.3.0) in R statistical software version 3.4.0. Briefly, reference lists (.cgi) were first converted to text (.txt) format. Then, abstracts were removed due to identical titles, using the find duplicates () function with method = exact, converting all title case to lowercase and removing punctuation. Additional references were removed using method = string_osa, optimal string alignment (restricted Damerau–Levenshtein distance), with a default threshold of 1, yielding a final reference list.

Abstracts were screened by two independent reviewers using Covidence [31]. Conflicts were resolved with internal discussion between the reviewers and in the case of a lasting conflict, by a third reviewer. Outcomes data and study demographics were extracted by two independent reviewers and data were consolidated into a final dataset by the two lead authors (MD and LRD).

### 2.3. Primary Outcomes

The co-primary endpoints of interest were overall survival (OS) and recurrence-free survival/disease-free survival (DFS). The definitions for these endpoints are described for each individual study in Table 1. Given that 9 out of 10 publications describe their endpoint as DFS rather than recurrence-free survival, DFS will be used to describe this endpoint.

### 2.4. Inclusion and Exclusion Criteria

Inclusion criteria were published reports of adult patients with a diagnosis of stage 2 or stage 3 CC with BRAF V600E mutation identified with a DNA-based assay and where the included study described either or both co-primary endpoints (DFS or OS) specifically for patients with BRAF mutations compared to BRAF WT. The results of this comparison must have been described as a univariate hazard ratio with 95% confidence interval (CI). Patients who received neoadjuvant systemic treatment and patients with rectal cancers were excluded. Studies that combined patients whose tumors harbored BRAF V600E with those whose tumors had BRAF non-V600 mutations were excluded given differential outcomes between these two groups [5]. Studies which exclusively compared BRAF V600E cancers to groups other than cohort-at-large BRAF WT tumors (such as a RAS WT subgroup) were excluded. Studies were also excluded if hazard ratios were derived from multivariate analyses.

### 2.5. Meta-Analysis

Meta-analysis was performed with a random-effects model that incorporated sample size, hazard ratio (HR), and 95% confidence intervals (CIs). Outcomes of interest included DFS and OS. Randomized controlled trials (RCTs) were pooled alone or together with retrospective studies for DFS and OS assessment. When possible, studies were also stratified by subgroup based on microsatellite instability-low/stable (MSS/MSI-L)/proficient mismatch repair (pMMR) and microsatellite instability-high/unstable (MSI/ MSI-H)/deficient mismatch repair (dMMR).

For a direct comparison between BRAF WT and BRAF-mutant patients, a restricted-maximum likelihood meta-analysis of HRs was performed. For each analysis, the 95% CIs, I^2^ statistic, and τ^2^ statistic were included. All tests were two-sided, and a *p* value  < 0.05 was considered significant unless otherwise specified. Analyses were performed using the metafor [32] and meta [33] packages in R statistical software version 3.4.0.

### 2.6. Quality Assessment

Sensitivity analyses were conducted for bias assessment. Leave-one-out meta-analyses were performed by omitting single studies and assessing their impact on overall estimates. We performed publication-bias assessments using funnel plots. Quality assessment of all included studies was performed using a modified Newcastle–Ottawa scale as previously described [34]. The six-point scale accounted for the following criteria: Selection—Did the patients represent all/consecutive colon cancer patients from the medical center/clinical trial? Ascertainment (Diagnosis)—Was the diagnosis correctly made with pathology-proven cancer and PCR or next-generation sequencing assays to confirm BRAF V600E mutation and exclude other BRAF mutations? Ascertainment (Outcome)—Was recurrence-free and overall survival ascertained? Follow-Up—Was follow-up long enough for treatment responses to be evaluated (1 year)? Reporting—Is the case described with sufficient details (e.g., drug posology, MSI/MMR status, stage, performance status, anatomical location of cancer) to allow other investigators to replicate the research or to allow practitioners make inferences related to their own practice? Tissue Collection—Was the tumor tissue collected in the context of a prospective trial? MINORS (Methological index for non-randomized studies) [35] was not used because the majority of the studies included in this meta-analysis are randomized clinical trials.

## 3. Results

### 3.1. Characteristics of Included Studies and Patients

**Figure 1:**
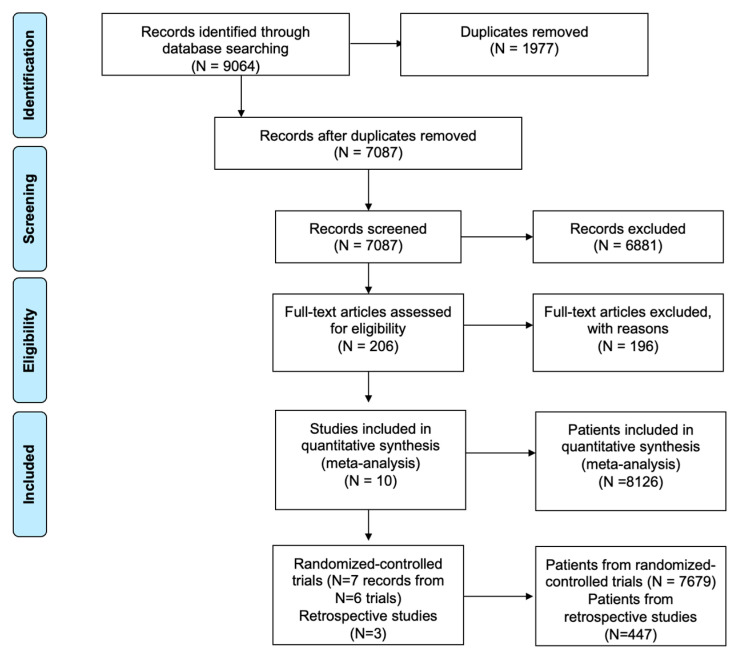
PRISMA diagram demonstrating search and inclusion of studies for meta-analysis.

The initial search yielded a total of 9064 results (Figure 1). After deduplication and title/abstract screening, 206 articles were assessed by full-text review. After removing ineligible articles, a total of ten articles were included in the review (Figure 1), comprising seven articles from six randomized controlled clinical trials (RCTs) [27,28,29,36,37,38,39], and three retrospective studies [40,41,42]. In total, 8126 patients were included in our analysis, of which 7679 (6836 BRAF WT, 843 BRAF V600E) were from RCTs and 447 (406 BRAF WT, 41 BRAF V600E) from retrospective studies (Table 1).

**Table 1:** Description of included studies. Abbreviations: RCT, randomized controlled trial; N, number; MSI, microsatellite instability; MMR, mismatch repair.

Study	Name of Study	Type of Study	Stage	N Total	N BRAF V600E	N BRAF Wild-Type	MSI/MMR Status Included	Adjuvant Treatment Regimen	Definition of OS Endpoint	Definition of RFS/DFS Endpoint
Taieb et al., 2016 [27]	PETACC-8	RCT	3	1643	148	1495	Yes	4 months of Oxaliplatin, Leucovorin, Fluorouracil +/− Cetuximab	Time from randomization until death from any cause.	DFS: time between randomization and local or metastatic recurrence, diagnosis of a second colon or rectal cancer, or death, whichever occurred first.
Sinicrope et al., 2015 [36]	NCCTG N0147	RCT	3	2831	346	2485	No	6 months of Oxaliplatin, Leucovorin, Fluorouracil +/− Cetuximab	Time from randomization until date of death; surviving patients were censored for OS at the date of last contact/follow-up. All patients were censored at 8 years post-randomization.	DFS: time from randomization until disease recurrence, or were censored at the date of last disease assessment if no recurrence occurred. For DFS, patients having died without recurrence were considered an event using date of death.
Sinicrope et al., 2013 [37]	NCCTG N0147	RCT	3	2480	340	2140	Yes	6 months of Oxaliplatin, Leucovorin, Fluorouracil +/− Cetuximab	Time from randomization until date of death; surviving patients were censored for OS at the date of last contact/follow-up. All patients were censored at 8 years post-randomization.	DFS: time from randomization until disease recurrence, or were censored at the date of last disease assessment if no recurrence occurred. For DFS, patients having died without recurrence were considered an event using date of death.
Roth et al., 2010 [28]	PETACC-3	RCT	2–3	1307	103	1204	Yes	6 months of Fluorouracil or Leucovorin +/− Irinotexan	Time from random assignment until death from any cause	RFS: time from the date of random assignment to the first date of local, regional, or distant relapse, the occurrence of a second primary colon cancer, or death.
Ogino et al., 2012 [38]	CALGB89803	RCT	3	506	75	431	No	8 months of Leucovorin and Fluorouracil +/− Irinotecan	Time from the study enrollment to death from any cause.	DFS: time from the study enrollment to cancer recurrence, occurrence of a new primary colon cancer, or death from any cause.
French et al., 2008 [39]	Intergroup 0135/NCCTG 91-46-53/NCIC CTG CO.9	RCT	2–3	490	77	413	Yes	Fluoruoracil and Leucovorin + high- vs. standard-dose Levamisole	Time from random assignment to the date of death or last contact. Censored at 8 years.	DFS: time from random assignment to the date of disease recurrence or death.
André et al., 2015 [29]	MOSAIC	RCT	2–3	902	94	808	No	6 months of Flurouracil or Leucovorin +/− Oxaliplatin	Time from random assignment to the date of death as a result of any cause.	DFS: time from random assignment to relapse or death, whichever occurred first. The occurrence of a second colorectal cancer was considered a relapse, whereas non-colorectal tumors were disregarded.
Sfakianaki et al., 2019 [40]	University of Crete	Retrospective	2–3	246	13	233	No	Fluorouracil or Capecitabine + Leucovorin and Oxaliplatin	Time from the date of surgery to date of death.	DFS: time between the date of colectomy to the first documented disease progression, second primary colon cancer, or death.
Küçükarda et al., 2022 [41]	Trakya University School of Medicine	Retrospective	1–3	23	11	12	No	Not described	Time between diagnosis and death for any cause.	DFS: time from the diagnosis to disease recurrence or development of distant metastasis.
Hu et al., 2020 [42]	Guangzhou Medical University	Retrospective	2–3	178	17	161	Yes	3 vs. 6 months of Oxaliplatin, Leucovorin and Fluorouracil	OS not reported, thus not defined.	DFS: time from surgery to the first event of local or metastatic recurrence, second primary cancer, or death from any cause.

**Figure 2:**
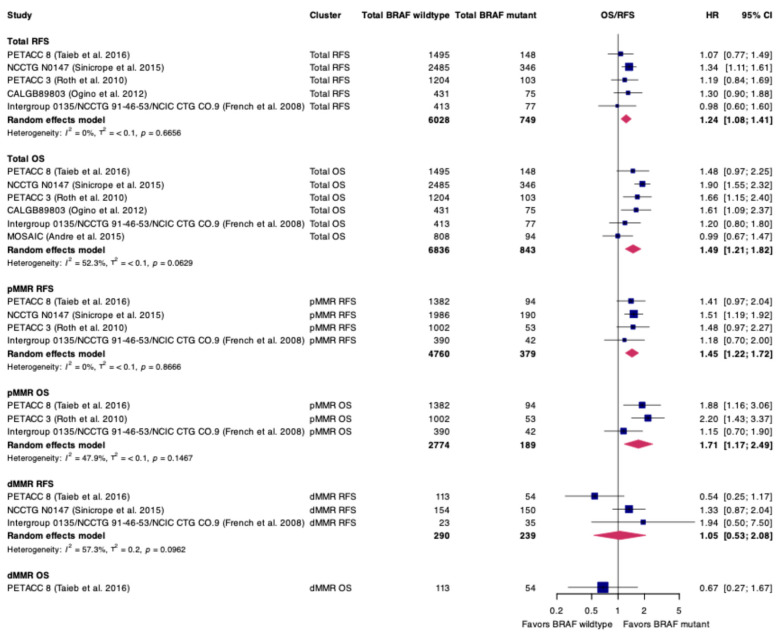
Pooled hazard ratios (HRs) of randomized controlled trials comparing BRAF V600E to BRAF wild-type (WT) early-stage colon cancer with the endpoints of disease-free survival (DFS) and overall survival (OS). CI: confidence interval, pMMR: proficient mismatch repair, MSS: microsatellite stable, dMMR: deficient mismatch repair, MSI-H: microsatellite instability-high. BRAF V600E-mutated early-stage CC patients have inferior outcomes compared to BRAF WT patients. Citations: PETACC-8 [27], NCCTG N0147 [36], PETACC-3 [28], CALGB89803 [38], Intergroup 0135/NCCTG 91-46-53/NCIC CTG CO.9 [39], MOSAIC [29].

Of the included studies, four included only patients with stage 3 disease, five included patients with stage 2–3 CC, and one included patients with stage 1–3 tumors (Table 1). A total of 5139 patients had pMMR/MSS tumors (4670 BRAF WT, 379 BRAF V600E) and 529 had dMMR/MSI-H tumors (290 BRAF WT, 239 BRAF V600E) (Figure 2, Table 1).

We next performed a meta-analysis of the included RCTs with endpoints of DFS and OS. We observed that patients whose tumors harbored BRAF V600E had significantly shorter DFS (HR 1.24, 95% CI 1.08–1.41; Figure 2). Patients whose tumors harbored BRAF V600E and were of dMMR/MSI-H status demonstrated no difference in DFS (HR 1.05, 95% CI 0.53–2.08). However, BRAF V600E-mutated patients with pMMR/MSS status similarly experienced inferior DFS compared to BRAF WT patients (HR 1.45, 95% CI 1.22–1.72).

Similar trends were observed when OS was used as the endpoint of interest. In the cohort at large, patients whose tumors harbored BRAF V600E mutations had inferior survival (HR 1.49, 95% CI 1.21–1.82). This finding similarly persisted in patients with pMMR/MSS (HR 1.71, 95% CI 1.17–2.49) but not dMMR/MSI-H, although only a single study was eligible for inclusion (HR 0.67, 95% CI 0.27–1.67).

When analyses were broadened to include retrospective studies, similar findings were observed. In the cohort at large, patients whose tumors harbored BRAF V600E experienced inferior DFS (HR 1.37, 95% CI 1.09–1.72) and OS (HR 1.55, 95% CI 1.30–1.85; Supplemental Figure S1). No additional patients were included from retrospective studies in the pMMR/MSS patient population, but an additional study of dMMR/MSI-H patients strengthened the null hypothesis of no-DFS-effect observed in these patients (HR 1.04, 95% CI 0.60–1.82) [42].

### 3.2. Quality Assessment

**Figure 3:**
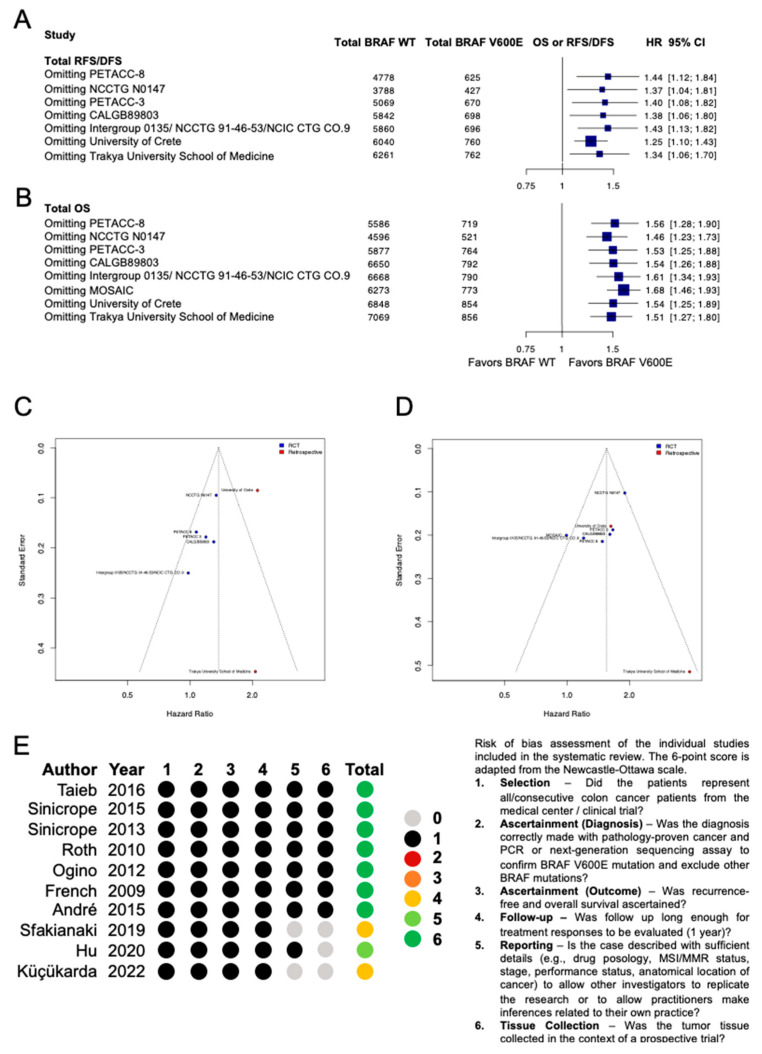
Quality assessment and risk-of-bias analysis. Pooled hazard ratios (HRs) of randomized controlled trials and retrospective studies comparing BRAF V600E to BRAF wild-type (WT) early-stage colon cancer in leave-one-out analysis with the endpoints of (**A**) disease-free survival (DFS) and (**B**) overall survival (OS). CI: confidence interval. (**C**) Funnel plot for publication bias in randomized controlled trials (RCTs) and retrospective studies with the endpoints of recurrence-free survival/disease-free survival and (**D**) overall survival. (**E**) Risk-of-bias assessment of the individual studies included in the systematic review. Citations: PETACC-8 [27], NCCTG N0147 [36,37], PETACC-3 [28], CALGB89803 [38], Intergroup 0135/NCCTG 91-46-53/NCIC CTG CO.9 [39], MOSAIC [29], University of Crete [40], Trakya University School of Medicine [41], Guangzhou Medical University [42].

To understand the context of the results described herein, an extensive quality assessment was performed. First, we performed sensitivity analyses with leave-one-out meta-analyses, which together demonstrate that if any included study is removed from DFS or OS analyses, the signal demonstrating that patients whose tumors harbor BRAF V600E mutations experience inferior prognosis remains (Figure 3A,B).

We next performed funnel plots to assess potential publication bias. On visual inspection of funnel plots for both DFS and OS, no overt evidence of publication bias is observed among randomized controlled trials (Figure 3C,D).

Quality assessment of all included studies was performed using a modified Newcastle–Ottawa scale. All six included RCTs fulfilled all six criteria (Figure 3E). Of the three retrospective studies included, all inherently lost one point for not being a randomized prospective study, and the Sfakianaki et al. University of Crete study lost an additional point for incomplete reporting of patient characteristics [40].

## 4. Discussion

Given renewed interest in early-stage BRAF V600E-mutant CC, we performed a systemic review and meta-analysis of this population to better understand the natural history of this entity relative to BRAF WT early-stage CC.

Two meta-analyses have been previously published on early-stage CRC patients with BRAF mutations. We were compelled to nonetheless perform this study for several reasons. Formica et al. meta-analyzed patients with BRAF-mutant stage 2 and 3 CC but included publications that used divergent statistical outcome measures [43,44], contained a subset of patients with rectal cancers which follow a different treatment algorithm altogether in the early-stage setting [45], and included patients with non-V600 BRAF mutations, which are known to be associated with differential prognosis and response to MAPK-targeted therapy [5,44,46]. Another meta-analysis by Zhu et al. similarly included publications that exclusively presented multivariate hazard ratios [43], patients with rectal cancers [47,48], and was published in 2016, resulting in an incomplete review of all currently available literature. In this study, we excluded rectal cancers and patients whose tumors harbored non-V600 BRAF mutations, closely reproducing the inclusion criteria of the ongoing A022004 study.

We found that BRAF V600E mutations confer a poor prognosis in early-stage CC in the population at large and in patients with MSS/MSI-L/pMMR tumors, but not MSI-H/dMMR tumors. This highlights MSS/MSI-L/pMMR BRAF-mutant early-stage CC patients as those poised to derive the most benefit from new treatment strategies, such as adjuvant-targeted therapy approaches.

The standard of care for early-stage MSS/pMMR CC includes chemotherapy with 5-fluorouracil with leucovorin and oxaliplatin (FOLFOX) or capecitabine and oxaliplatin (CAPOX) [49]. For the high-risk stage 2 or stage 3 patients included in the A022004/NCT05710406 study with MSS/pMMR, standard of care in the adjuvant setting includes 3–6 months of CAPOX or 6 months of FOLFOX chemotherapy [49]. However, it remains unclear whether either of these regimens, or a different chemotherapy regimen altogether, is superior for early-stage CC with BRAF V600E mutations.

Beyond MAPK-targeted therapies, immune-checkpoint inhibition (ICI) may also play a prominent role in the management of metastatic CRC with Class 1 BRAF mutations. Indeed, 20–25% of metastatic CRC with BRAF V600E have concurrent microsatellite instability-high (MSI-H)/deficient mismatch repair (dMMR) [50,51], which are known biomarkers predicting response to ICI [51]. The ongoing SEAMARK trial aims to study the combination of encorafenib, cetuximab, and pembrolizumab in the first line for metastatic CRC with MSI-H/dMMR and BRAF V600E [52].

There are several important limitations associated with this study. Given that the data in the included studies are described in aggregate and exclusively use hazard ratios to compare groups without providing median survival times, there is insufficient information to establish a consensus on expected DFS and OS times for these patients. Accessing individual-patient data to better delineate survival times for these patients may prove helpful in the clinical-trial design of studies for this patient population. For the same reason, this study was unable to determine whether there were differences in efficacy across various adjuvant chemotherapy regimens or duration of adjuvant therapy for the BRAF-mutant subset.

The ongoing, highly anticipated A022004 randomized trial is enrolling patients with stage 2/3 BRAF V600E-mutant CC to evaluate treatment with encorafenib and cetuximab [22]. This meta-analysis provides important context for understanding the natural history of the patient subgroup included in this trial and will serve as a useful reference for interpreting its forthcoming results. In addition, the findings of this meta-analysis aim to guide the design of future studies in this setting.

## Data Availability

All data were sourced from previously published randomized controlled trials.

## Supplementary Materials

The following supporting information can be downloaded at: https://www.mdpi.com/article/10.3390/curroncol32110624/s1, Figure S1: Pooled hazard ratios (HR) of randomized-controlled trials and retrospective studies comparing BRAF V600E to BRAF wild-type (WT) early-stage colon cancer with the endpoints of disease-free survival (DFS) and overall survival (OS). CI: confidence interval, pMMR: proficient mismatch repair, MSS: microsatellite stable, dMMR: deficient mismatch repair, MSI-H: microsatellite instability high.

## Abbreviations

The following abbreviations are used in this manuscript:

CC	Colon cancer
OS	Overall survival
DFS	Disease-free survival
HR	Hazard ratio
WT	Wild-type
ORR	Overall response rate

## References

[1] Dankner M., Rose A.A.N., Rajkumar S., Siegel P.M., Watson I.R. (2018). Classifying BRAF alterations in cancer: New rational therapeutic strategies for actionable mutations. Oncogene.

[2] Kazandjian S., Rousselle E., Dankner M., Cescon D.W., Spreafico A., Ma K., Kavan P., Batist G., Rose A.A.N. (2024). The Clinical, Genomic, and Transcriptomic Landscape of BRAF Mutant Cancers. Cancers.

[3] Dankner M. (2018). Targeted Therapy for Colorectal Cancers With Non-V600 BRAF Mutations: Perspectives for Precision Oncology. JCO Precis. Oncol..

[4] Dankner M., Wang Y., Fazelzad R., Johnson B., Nebhan C.A., Dagogo-Jack I., Myall N.J., Richtig G., Bracht J.W.P., Gerlinger M. (2022). Clinical Activity of Mitogen-Activated Protein Kinase-Targeted Therapies in Patients With Non-V600 BRAF-Mutant Tumors. JCO Precis. Oncol..

[5] Jones J.C., Renfro L.A., Al-Shamsi H.O., Schrock A.B., Rankin A., Zhang B.Y., Kasi P.M., Voss J.S., Leal A.D., Sun J. (2017). (Non-V600) BRAF Mutations Define a Clinically Distinct Molecular Subtype of Metastatic Colorectal Cancer. J. Clin. Oncol..

[6] Hauschild A., Grob J.J., Demidov L.V., Jouary T., Gutzmer R., Millward M., Rutkowski P., Blank C.U., Miller W.H., Kaempgen E. (2012). Dabrafenib in BRAF-mutated metastatic melanoma: A multicentre, open-label, phase 3 randomised controlled trial. Lancet.

[7] Sosman J.A., Kim K.B., Schuchter L., Gonzalez R., Pavlick A.C., Weber J.S., McArthur G.A., Hutson T.E., Moschos S.J., Flaherty K.T. (2012). Survival in BRAF V600-mutant advanced melanoma treated with vemurafenib. N. Engl. J. Med..

[8] Chapman P.B., Hauschild A., Robert C., Haanen J.B., Ascierto P., Larkin J., Dummer R., Garbe C., Testori A., Maio M. (2011). Improved survival with vemurafenib in melanoma with BRAF V600E mutation. N. Engl. J. Med..

[9] Flaherty K.T., Infante J.R., Daud A., Gonzalez R., Kefford R.F., Sosman J., Hamid O., Schuchter L., Cebon J., Ibrahim N. (2012). Combined BRAF and MEK inhibition in melanoma with BRAF V600 mutations. N. Engl. J. Med..

[10] Larkin J., Ascierto P.A., Dréno B., Atkinson V., Liszkay G., Maio M., Mandalà M., Demidov L., Stroyakovskiy D., Thomas L. (2014). Combined vemurafenib and cobimetinib in BRAF-mutated melanoma. N. Engl. J. Med..

[11] Kopetz S., Desai J., Chan E., Hecht J.R., O’Dwyer P.J., Maru D., Morris V., Janku F., Dasari A., Chung W. (2015). Phase II Pilot Study of Vemurafenib in Patients With Metastatic BRAF-Mutated Colorectal Cancer. J. Clin. Oncol..

[12] Hyman D.M., Puzanov I., Subbiah V., Faris J.E., Chau I., Blay J.Y., Wolf J., Raje N.S., Diamond E.L., Hollebecque A. (2015). Vemurafenib in Multiple Nonmelanoma Cancers with BRAF V600 Mutations. N. Engl. J. Med..

[13] Corcoran R.B., Ebi H., Turke A.B., Coffee E.M., Nishino M., Cogdill A.P., Brown R.D., Della Pelle P., Dias-Santagata D., Hung K.E. (2012). EGFR-mediated re-activation of MAPK signaling contributes to insensitivity of BRAF mutant colorectal cancers to RAF inhibition with vemurafenib. Cancer Discov..

[14] Prahallad A., Sun C., Huang S., Di Nicolantonio F., Salazar R., Zecchin D., Beijersbergen R.L., Bardelli A., Bernards R. (2012). Unresponsiveness of colon cancer to BRAF(V600E) inhibition through feedback activation of EGFR. Nature.

[15] Yaeger R., Cercek A., O’Reilly E.M., Reidy D.L., Kemeny N., Wolinsky T., Capanu M., Gollub M.J., Rosen N., Berger M.F. (2015). Pilot trial of combined BRAF and EGFR inhibition in BRAF-mutant metastatic colorectal cancer patients. Clin. Cancer Res..

[16] Kopetz S., Guthrie K.A., Morris V.K., Lenz H.J., Magliocco A.M., Maru D., Yan Y., Lanman R., Manyam G., Hong D.S. (2021). Randomized Trial of Irinotecan and Cetuximab With or Without Vemurafenib in BRAF-Mutant Metastatic Colorectal Cancer (SWOG S1406). J. Clin. Oncol..

[17] Kopetz S., Grothey A., Yaeger R., Van Cutsem E., Desai J., Yoshino T., Wasan H., Ciardiello F., Loupakis F., Hong Y.S. (2019). Encorafenib, Binimetinib, and Cetuximab in BRAF V600E-Mutated Colorectal Cancer. N. Engl. J. Med..

[18] Tabernero J., Grothey A., Van Cutsem E., Yaeger R., Wasan H., Yoshino T., Desai J., Ciardiello F., Loupakis F., Hong Y.S. (2021). Encorafenib Plus Cetuximab as a New Standard of Care for Previously Treated BRAF V600E-Mutant Metastatic Colorectal Cancer: Updated Survival Results and Subgroup Analyses from the BEACON Study. J. Clin. Oncol..

[19] Van Cutsem E., Taieb J., Yaeger R., Yoshino T., Grothey A., Maiello E., Elez E., Dekervel J., Ross P., Ruiz-Casado A. (2023). ANCHOR CRC: Results From a Single-Arm, Phase II Study of Encorafenib Plus Binimetinib and Cetuximab in Previously Untreated BRAF(V600E)-Mutant Metastatic Colorectal Cancer. J. Clin. Oncol..

[20] Kopetz S., Yoshino T., Van Cutsem E., Eng C., Kim T.W., Wasan H.S., Desai J., Ciardiello F., Yaeger R., Maughan T.S. (2025). Encorafenib, cetuximab and chemotherapy in BRAF-mutant colorectal cancer: A randomized phase 3 trial. Nat. Med..

[21] Boku S., Satake H., Ohta T., Mitani S., Kawakami K., Suzuki Y., Matsumoto T., Terazawa T., Yamazaki E., Hasegawa H. (2022). TRESBIEN (OGSG 2101): Encorafenib, binimetinib and cetuximab for early recurrent stage II/III BRAF V600E-mutated colorectal cancer. Future Oncol..

[22] Yaeger R., Shi Q., Dueck A.C., Dib E.G., Kazmi S.M.A., Alese O.B., Krishnamurthi S.S., Nixon A.B., Shergill A., O’Reilly E.M. (2023). A randomized trial of consolidation-targeted adjuvant therapy with encorafenib and cetuximab versus usual care for patients with stage II/III BRAF V600E colon cancer: Alliance for Clinical Trials in Oncology A022004. J. Clin. Oncol..

[23] Ciappina G., Toscano E., Ottaiano A., Capuozzo M., Consolo P., Maiorana E., Carroccio P., Franchina T., Ieni A., Di Mauro A. (2025). Negative Hyperselection in Metastatic Colorectal Cancer for First-Line Anti-EGFR Therapy: A Narrative Review. Int. J. Mol. Sci..

[24] Yaeger R., Cercek A., Chou J.F., Sylvester B.E., Kemeny N.E., Hechtman J.F., Ladanyi M., Rosen N., Weiser M.R., Capanu M. (2014). BRAF mutation predicts for poor outcomes after metastasectomy in patients with metastatic colorectal cancer. Cancer.

[25] Richman S.D., Seymour M.T., Chambers P., Elliott F., Daly C.L., Meade A.M., Taylor G., Barrett J.H., Quirke P. (2009). KRAS and BRAF mutations in advanced colorectal cancer are associated with poor prognosis but do not preclude benefit from oxaliplatin or irinotecan: Results from the MRC FOCUS trial. J. Clin. Oncol..

[26] Clarke C.N., Kopetz E.S. (2015). BRAF mutant colorectal cancer as a distinct subset of colorectal cancer: Clinical characteristics, clinical behavior, and response to targeted therapies. J. Gastrointest. Oncol..

[27] Taieb J., Zaanan A., Le Malicot K., Julié C., Blons H., Mineur L., Bennouna J., Tabernero J., Mini E., Folprecht G. (2016). Prognostic Effect of BRAF and KRAS Mutations in Patients With Stage III Colon Cancer Treated With Leucovorin, Fluorouracil, and Oxaliplatin With or Without Cetuximab: A Post Hoc Analysis of the PETACC-8 Trial. JAMA Oncol..

[28] Roth A.D., Tejpar S., Delorenzi M., Yan P., Fiocca R., Klingbiel D., Dietrich D., Biesmans B., Bodoky G., Barone C. (2010). Prognostic role of KRAS and BRAF in stage II and III resected colon cancer: Results of the translational study on the PETACC-3, EORTC 40993, SAKK 60-00 trial. J. Clin. Oncol..

[29] André T., de Gramont A., Vernerey D., Chibaudel B., Bonnetain F., Tijeras-Raballand A., Scriva A., Hickish T., Tabernero J., Van Laethem J.L. (2015). Adjuvant Fluorouracil, Leucovorin, and Oxaliplatin in Stage II to III Colon Cancer: Updated 10-Year Survival and Outcomes According to BRAF Mutation and Mismatch Repair Status of the MOSAIC Study. J. Clin. Oncol..

[30] Moher D., Liberati A., Tetzlaff J., Altman D.G. (2009). Preferred reporting items for systematic reviews and meta-analyses: The PRISMA statement. Ann. Intern. Med..

[31] Babineau J. (2014). Product Review: Covidence (Systematic Review Software). J. Can. Health Libr. Assoc./J. L’association Bibliothèques Santé Can..

[32] Viechtbauer W. (2010). Conducting Meta-Analyses in R with the metafor Package. J. Stat. Softw..

[33] Balduzzi S., Rücker G., Schwarzer G. (2019). How to perform a meta-analysis with R: A practical tutorial. Evid. Based Ment. Health.

[34] Lazaratos A.M., Maritan S.M., Quaiattini A., Darlix A., Ratosa I., Ferraro E., Griguolo G., Guarneri V., Pellerino A., Hofer S. (2023). Intrathecal trastuzumab versus alternate routes of delivery for HER2-targeted therapies in patients with HER2+ breast cancer leptomeningeal metastases. Breast.

[35] Slim K., Nini E., Forestier D., Kwiatkowski F., Panis Y., Chipponi J. (2003). Methodological index for non-randomized studies (minors): Development and validation of a new instrument. ANZ J. Surg..

[36] Sinicrope F.A., Mahoney M.R., Yoon H.H., Smyrk T.C., Thibodeau S.N., Goldberg R.M., Nelson G.D., Sargent D.J., Alberts S.R. (2015). Analysis of Molecular Markers by Anatomic Tumor Site in Stage III Colon Carcinomas from Adjuvant Chemotherapy Trial NCCTG N0147 (Alliance). Clin. Cancer Res..

[37] Sinicrope F.A., Mahoney M.R., Smyrk T.C., Thibodeau S.N., Warren R.S., Bertagnolli M.M., Nelson G.D., Goldberg R.M., Sargent D.J., Alberts S.R. (2013). Prognostic impact of deficient DNA mismatch repair in patients with stage III colon cancer from a randomized trial of FOLFOX-based adjuvant chemotherapy. J. Clin. Oncol..

[38] Ogino S., Shima K., Meyerhardt J.A., McCleary N.J., Ng K., Hollis D., Saltz L.B., Mayer R.J., Schaefer P., Whittom R. (2012). Predictive and prognostic roles of BRAF mutation in stage III colon cancer: Results from intergroup trial CALGB 89803. Clin. Cancer Res..

[39] French A.J., Sargent D.J., Burgart L.J., Foster N.R., Kabat B.F., Goldberg R., Shepherd L., Windschitl H.E., Thibodeau S.N. (2008). Prognostic significance of defective mismatch repair and BRAF V600E in patients with colon cancer. Clin. Cancer Res..

[40] Sfakianaki M., Papadaki C., Tzardi M., Trypaki M., Alam S., Lagoudaki E.D., Messaritakis I., Zoras O., Mavroudis D., Georgoulias V. (2019). Loss of LKB1 Protein Expression Correlates with Increased Risk of Recurrence and Death in Patients with Resected, Stage II or III Colon Cancer. Cancer Res. Treat..

[41] Küçükarda A., Gökyer A., Sayın S., Gökmen İ., Özcan E., Köstek O., Hacıoğlu M.B., Uzunoğlu S., Çiçin İ., Erdoğan B. (2022). Prognostic Factors for Survival in Transverse Colon Cancers. J. Gastrointest. Cancer.

[42] Hu H., Wu Z., Wang C., Huang Y., Zhang J., Cai Y., Xie X., Li J., Shen C., Li W. (2020). Duration of FOLFOX Adjuvant Chemotherapy in High-Risk Stage II and Stage III Colon Cancer With Deficient Mismatch Repair. Front. Oncol..

[43] Gavin P.G., Colangelo L.H., Fumagalli D., Tanaka N., Remillard M.Y., Yothers G., Kim C., Taniyama Y., Kim S.I., Choi H.J. (2012). Mutation profiling and microsatellite instability in stage II and III colon cancer: An assessment of their prognostic and oxaliplatin predictive value. Clin. Cancer Res..

[44] Hutchins G., Southward K., Handley K., Magill L., Beaumont C., Stahlschmidt J., Richman S., Chambers P., Seymour M., Kerr D. (2011). Value of mismatch repair, KRAS, and BRAF mutations in predicting recurrence and benefits from chemotherapy in colorectal cancer. J. Clin. Oncol..

[45] Domingo E., Camps C., Kaisaki P.J., Parsons M.J., Mouradov D., Pentony M.M., Makino S., Palmieri M., Ward R.L., Hawkins N.J. (2018). Mutation burden and other molecular markers of prognosis in colorectal cancer treated with curative intent: Results from the QUASAR 2 clinical trial and an Australian community-based series. Lancet Gastroenterol. Hepatol..

[46] Formica V., Sera F., Cremolini C., Riondino S., Morelli C., Arkenau H.T., Roselli M. (2022). KRAS and BRAF Mutations in Stage II and III Colon Cancer: A Systematic Review and Meta-Analysis. J. Natl. Cancer Inst..

[47] Zhu L., Dong C., Cao Y., Fang X., Zhong C., Li D., Yuan Y. (2016). Prognostic Role of BRAF Mutation in Stage II/III Colorectal Cancer Receiving Curative Resection and Adjuvant Chemotherapy: A Meta-Analysis Based on Randomized Clinical Trials. PLoS ONE.

[48] Pentheroudakis G., Raptou G., Kotoula V., Wirtz R.M., Vrettou E., Karavasilis V., Gourgioti G., Gakou C., Syrigos K.N., Bournakis E. (2015). Immune response gene expression in colorectal cancer carries distinct prognostic implications according to tissue, stage and site: A prospective retrospective translational study in the context of a hellenic cooperative oncology group randomised trial. PLoS ONE.

[49] Argilés G., Tabernero J., Labianca R., Hochhauser D., Salazar R., Iveson T., Laurent-Puig P., Quirke P., Yoshino T., Taieb J. (2020). Localised colon cancer: ESMO Clinical Practice Guidelines for diagnosis, treatment and follow-up. Ann. Oncol..

[50] Venderbosch S., Nagtegaal I.D., Maughan T.S., Smith C.G., Cheadle J.P., Fisher D., Kaplan R., Quirke P., Seymour M.T., Richman S.D. (2014). Mismatch repair status and BRAF mutation status in metastatic colorectal cancer patients: A pooled analysis of the CAIRO, CAIRO2, COIN, and FOCUS studies. Clin. Cancer Res..

[51] André T., Shiu K.K., Kim T.W., Jensen B.V., Jensen L.H., Punt C., Smith D., Garcia-Carbonero R., Benavides M., Gibbs P. (2020). Pembrolizumab in Microsatellite-Instability-High Advanced Colorectal Cancer. N. Engl. J. Med..

[52] Elez E., Kopetz S., Tabernero J., Bekaii-Saab T., Taieb J., Yoshino T., Manji G., Fernandez K., Abbattista A., Zhang X. (2023). SEAMARK: Phase II study of first-line encorafenib and cetuximab plus pembrolizumab for MSI-H/dMMR BRAFV600E-mutant mCRC. Future Oncol..

