# Supplementary material for: Defining the Prognostic Significance of BRAF V600E in Early-Stage Colon Cancer: A Systematic Review and Meta-Analysis
Source: Curr Oncol. 2025 Nov 6;32(11):624. doi: 10.3390/curroncol32110624 (PMC12651658; doi:10.3390/curroncol32110624)
Supplement: Supplementary file 1 [file curroncol-32-00624-s001.zip › curroncol-3811807-supplementary.pdf]

Supplemental Material

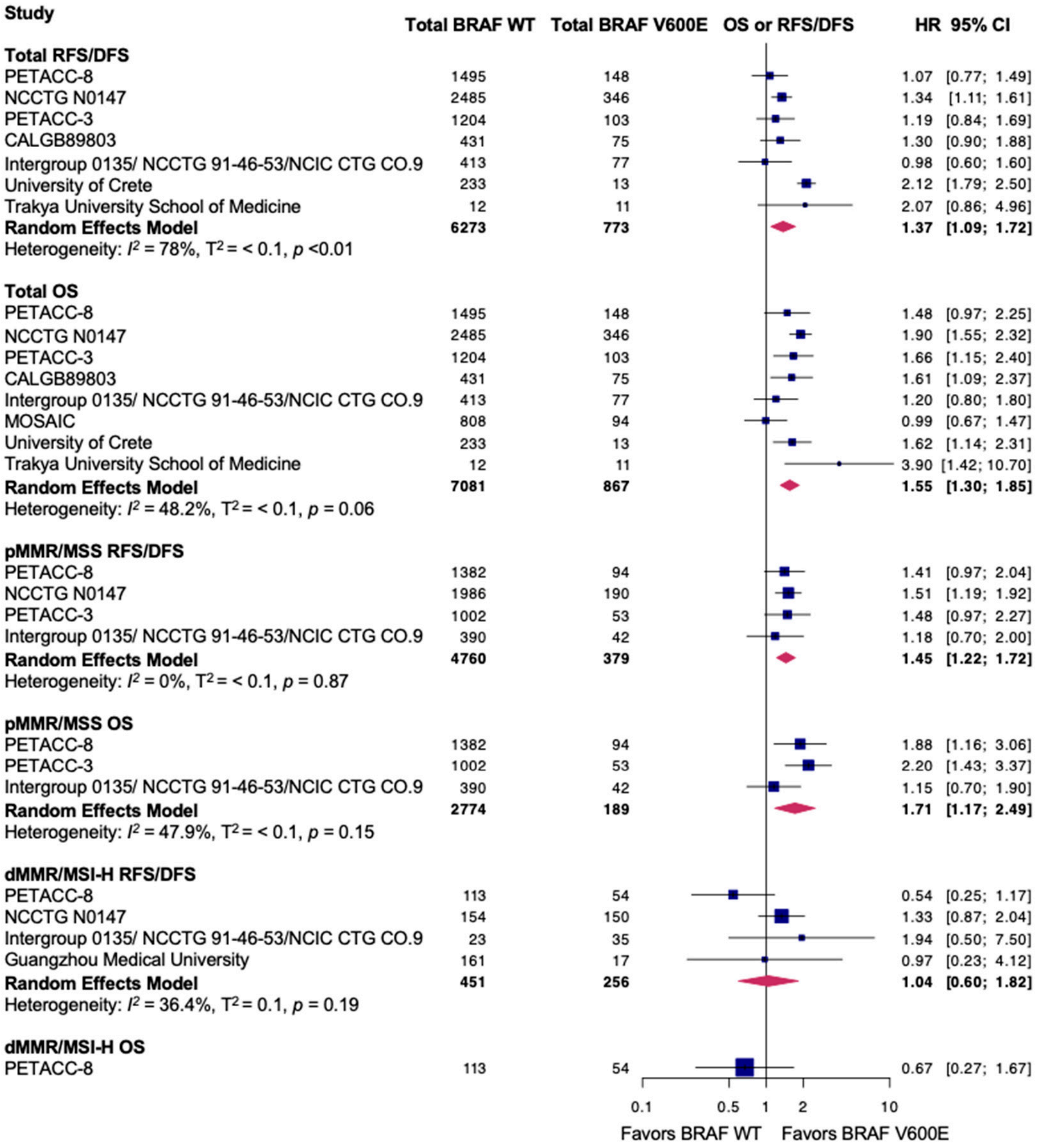

Figure S1. Pooled hazard ratios (HR) of randomized-controlled trials and retrospective studies comparing BRAF V600E to BRAF wild-type (WT) early-stage colon cancer with the endpoints of disease-free survival (DFS) and overall survival (OS). CI: confidence interval, pMMR: proficient mismatch repair, MSS: microsatellite stable, dMMR: deficient mismatch repair, MSI-H: microsatellite instability high.
